# Germinoma with Diffuse Subependymal Spread: A Case Report

**DOI:** 10.7759/cureus.643

**Published:** 2016-06-15

**Authors:** Evan M Krueger, Darbi L Invergo, Julian J Lin

**Affiliations:** 1 Neurosurgery, Advocate Health Care; 2 Neurosurgery, University of Illinois College of Medicine at Peoria

**Keywords:** intracranial germinoma, diffuse subependymal, ventricles, suprasellar, pineal

## Abstract

A 19-year-old Caucasian male presented with complaints of headaches and syncope. Suspicion of hydrocephalus prompted computed tomography (CT) and magnetic resonance imaging (MRI), which revealed pineal and suprasellar prominences with diffuse, thick, nodular subependymal enhancement of the lateral and third ventricles. Based on imaging, the differential diagnosis consisted primarily of malignancy, such as lymphoma, with inflammatory and infectious etiologies not excluded. Cerebrospinal fluid (CSF) samples were non-specific, and neuroendoscopic tissue biopsy histologically confirmed the diagnosis of pure germinoma. The patient was treated with radiation, and follow-up MRIs at one, three, six, and 12 months demonstrated progressive resolution of tumor burden with marked clinical improvement.

Germinomas are rare germ cell tumors that are more frequently diagnosed in Asian countries. They uncommonly seed into the lateral ventricles, and only two other cases have been described with diffuse subependymal involvement. Unlike other malignant germ cell tumors, germinomas have marker negative CSF samples that are important in the normal diagnostic workup of diffuse subependymal lesions. Histopathologic correlation is required for definitive diagnosis in the United States and can be achieved with endoscopic tissue sampling. Germinomas are highly radio- and chemotherapy sensitive and have a fair prognosis with modern therapeutic techniques. Germinoma should be considered with simultaneous midline and diffuse ventricular lesions.

## Introduction

The World Health Organization (WHO) categorizes central nervous system (CNS) germ cell tumors (GCTs) as non-germinomas and germinomas [1]. While rare, germinomas are the most common GCT composing 36 - 70% of all cases [2-10]. The incidence of CNS GCTs varies significantly according to geography, accounting for 0.1 - 3.4% of all primary brain tumors in Western countries [2-3, 6, 11-14]. In the United States, the estimated incidence of intracranial germinoma is 0.1/100,000 persons [15], and in Canada, the incidence is 1.06/1,000,000 for those less than 19 years old [3]. In Asian countries, the incidence of CNS GCTs dramatically increases to between 4.8% and 15% of intracranial neoplasms [6, 16-22]. Germinomas have a male to female ratio of 1.8 - 3.5:1, and are diagnosed at a mean age of 11.6 - 12.3 years old [3, 6].

Clinical presentation is variable and dictated by tumor location. A careful history and physical exam detailing any endocrine, circadian rhythm, and cranial nerve abnormalities, as well as symptoms of increased intracranial pressure, is helpful. Germinomas typically present in the pineal (38 - 57%) or suprasellar regions (34 - 49%), less frequently as a double lesion in the pineal and suprasellar regions synchronously (5-10%), and rarely in other locations (3-5%), including the ventricles [2-3, 6, 23-25]. GCTs have inconsistent radiographic appearances [3, 26-29]. A retrospective analysis of 18 separate cases of germinomas showed all tumors had solid components that enhanced homogeneously in eight (44%) cases and heterogeneously in 10 (56%) cases while eight (44%) cases had cystic components [28].

## Case presentation

**Figure 1:**
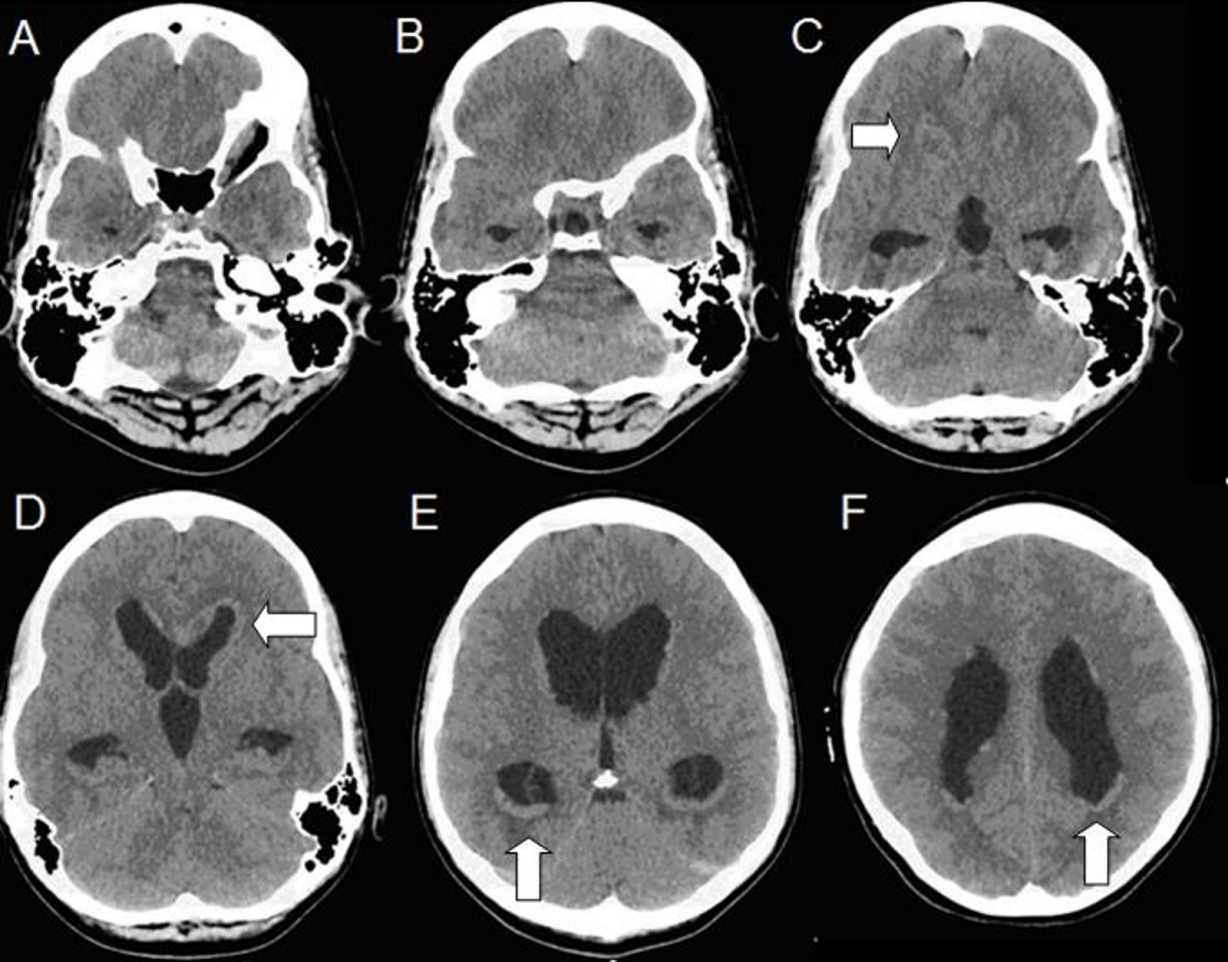
Non-contrast-enhanced CT head at initial presentation. There is transependymal fluid shift with enlargement of the lateral and third ventricles with nodular densities prominent in the occipital and anterior horns bilaterally.

A 19-year-old Caucasian male presented to an outside emergency department with a variety of non-specific symptoms, including severe headaches with resultant emesis and syncopal episodes. A review of systems was otherwise essentially negative for constitutional, neurological, and endocrine symptoms. There was no history of prior meningitis, frequent infections, familial cancers, or high-risk behaviors, such as illicit drug usage. The patient's vitals were stable and extensive lab workup was normal, but fundoscopic examination showed optic pallor bilaterally. A computed tomography scan (CT) was ordered for suspected hydrocephalus, which demonstrated a transependymal fluid shift with enlargement of the lateral and third ventricles with nodular densities prominent in the occipital and anterior horns bilaterally (Figure 1). Informed patient consent was obtained for treatment.

A right occipital ventriculoperitoneal shunt was placed and cerebrospinal fluid (CSF) samples were sent for cytology with the suspicion of an underlying malignancy, such as lymphoma, inflammatory, or infectious etiology. CSF samples showed a non-specific reactive T-cell lymphocytosis but were otherwise unremarkable for tumor and inflammatory markers, including for alpha-fetoprotein (AFP) and beta-human chorionic gonadotropin (β-hCG).

**Figure 2:**
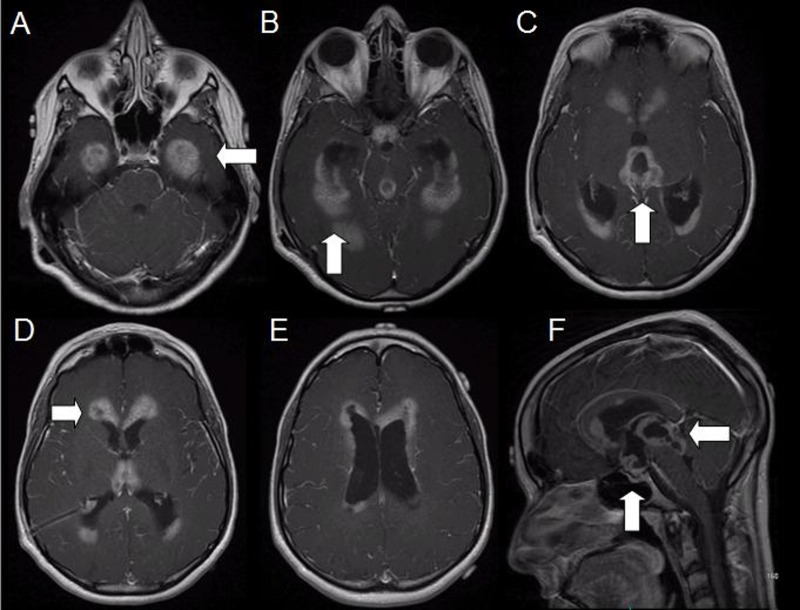
Post-contrast axial and sagittal MRI sequences with neuronavigation protocol at diagnosis. There is diffuse involvement of the lateral and third ventricles, the suprasellar and pineal regions, and the aqueduct with resultant ventriculomegaly.

The patient was then evaluated at a tertiary center where a right frontal endoscopic biopsy was undertaken for definitive diagnosis. Preoperative magnetic resonance imaging (MRI) confirmed extensive subependymal enhancement in a thick, nodular pattern diffusely throughout both lateral ventricles and the pineal region as well as the diffuse involvement of the third ventricle, including the foramen of Monro, suprasellar region, and Sylvian aqueduct, with moderate ventricular and aqueduct dilatation (Figure 2).

Tissue samples taken from the right frontal horn of the lateral ventricle were once again negative for AFP and β-hCG but positive for cytoplasmic placental-like alkaline phosphatase (PLAP), confirming the diagnosis of pure CNS germinoma.

**Figure 3:**
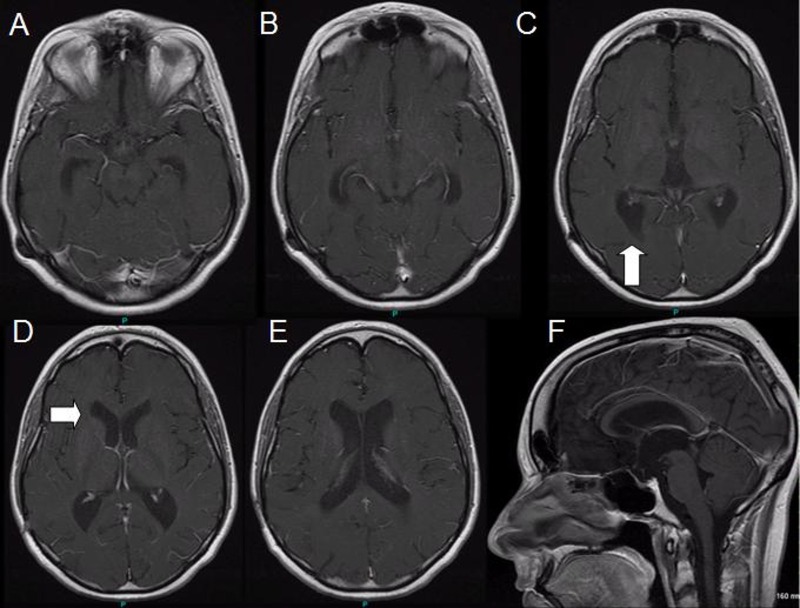
Post-contrast axial and sagittal MRI sequences one year after diagnosis and initiation of radiation therapy. There is resolution of tumor burden with mild residual ventriculomegaly.

The patient was treated with 25.4 Gy radiation to the cranial-spinal axis due to the significant intracranial spread, 36 Gy total whole brain radiation, and 50.4 Gy (in 1.8 Gy or fewer fractions) boosts to the prominent midline and ventricular regions. Follow-up MRIs at one, three, six, and 12 months demonstrated diminishing tumor burden with mild residual lateral ventriculomegaly (Figure 3).

At his last visit, the patient was asymptomatic with a resolution of his syncopal episodes and headaches.

## Discussion

This patient’s imaging characteristics were not typical of any particular lesion; therefore, neoplastic, inflammatory, and infectious etiologies all needed to be considered. For the patient’s age and diffuse subependymal involvement of the lateral and third ventricles, lymphoma and metastasis were the main concern. The differential for masses of the ventricular system is vast, and when including the pineal and suprasellar regions as well, the possibilities are immense [30-32]. Germinoma was a far less likely possibility, given the geographic presentation, radiographic appearance, and a few cases in the literature of diffuse subependymal involvement [29, 33-34].

CNS GCTs are categorized based on secreted tumor markers that are most sensitive and reliably measured in CSF [1, 35]. Yolk sac tumors and choriocarcinomas often present with AFP and β-hCG elevation, respectively, while the marker negative CSF samples obtained in our case were suggestive of a diagnosis of germinoma [36]. Current screening controversies surround the sensitivity of CSF sampling and whether ≤ 50 mIU/L CSF β-hCG or ≤ 200 mIU/L CSF β-hCG should be suggestive of a pure germinoma [26]. While CSF sampling and radiographic imaging are helpful, GCTs ultimately require tissue sampling for diagnosis in the United States [26, 29, 37]. Tissues that stain positive for PLAP is pathognomonic for germinoma.

Intracranial germinomas are highly curable tumors with a fair prognosis despite being WHO Grade IV lesions and rapidly fatal, if untreated [1]. Radiation and chemotherapy results in a five-year progression-free survival of 83 - 100% and an overall survival of 89 - 100% [1, 38-39]. A nonrandomized international study for intracranial germinomas comparing craniospinal irradiation versus chemotherapy, plus local radiotherapy, showed there were no differences in five-year event-free overall survival, although the radiotherapy alone arm had improved progression-free survival [40]. The optimal treatment modality remains controversial, and future directions will continue to focus on balancing adverse effects with curative goals [26].

A review of the English literature demonstrated only 17 other reported cases of a germinoma in the lateral ventricles, with a majority of these cases occurring in Asian countries. However, many of these cases had involvement of the ventricles only (Table 1A) [23, 41-44], a presumed midline epicenter involving the lateral ventricles only (Table 1B) [42, 45], reoccurrence along a shunt tube (Table 1C) [46], a presumed midline epicenter with non-diffuse involvement of one or multiple ventricles (Table 1D) [42, 47-49], or a non-pure germinoma with diffuse subependymal involvement (Table 1E) [29]. In 2012, Chen, et al. described their therapeutic experiences treating disseminated germinomas in an Asian country and mentioned two cases involving seeding in multiple ventricles [39]. Although the differences were subtle, we were only able to find two other cases in the literature of a pure intracranial germinoma with extensive seeding of the lateral and third ventricles; however, those cases also involved extensive metastasis resulting in death (Table 1F) [33-34]. Therefore, this case (Table 1G) importantly represents an exceedingly rare occurrence in a Western country of a potentially curable neoplasm that should be included in a differential diagnosis in cases of midline anomalies with diffuse subependymal involvement.

**Table 1:** English Literature Germinoma in the Lateral Ventricles Group A: Germinoma in lateral and other ventricles. Group B: Germinoma in lateral ventricles only with midline epicenter. Group C: Germinoma recurrence along shunt tract. Group D: Germinoma with midline epicenter and non-diffuse involvement of one or multiple ventricles. Group E: Non-pure germinoma with diffuse subependymal involvement. Group F: Pure germinoma with diffuse seeding in lateral and third ventricles resulting in death. Group G: present case.

Group	Reference	Age, Sex	Race	Country	Imaging	Primary	Dissemination	Outcome
A	[44]	19, M	-	Japan	Heterogeneous, Enhancing	Septum Pellucidum	Left Lateral Ventricle, Basal Ganglia	-
A	[41]	-	-	South Korea	-	-	Lateral and Third Ventricles	-
A	[42]	28, M	-	Japan	-	Lateral Ventricle	-	No reoccurrence at 13 years
A	[42]	30, M	-	Japan	-	Lateral Ventricle	Third and Fourth Ventricles	No reoccurrence at 7 years
A	[23]	13, M	-	South Korea	-	Septum	Frontal Horn Lateral Ventricle	No reoccurrence at 10 years
A	[43]	25, F	-	Taiwan	Enhancing	Right Frontal Horn Lateral Ventricle	Septum Pellucidum	No reoccurrence at 6 months
B	[42]	18, M	-	Japan	-	Pineal Gland	Lateral Ventricle	No reoccurrence at 13 years
B	[45]	27, M	-	Japan	Low-intensity T1, High-intensity T2, Enhancing	Intraparenchymal	Lateral Ventricle	No reoccurrence at 1 year
C	[46]	38, M	-	Japan	-	Suprasellar	Lateral Ventricle	Reoccurrence at 5 years, twice between years 6 and 7
D	[48]	23, M	Caucasian	Germany	Circumscribed, Multifocal, Homogenously Enhancing	Midline	Anterior Horns Lateral Ventricles	No reoccurrence at 3 months
D	[42]	18, M	-	Japan	-	Suprasellar	Basal Ganglia, Lateral Ventricle	No reoccurrence at 6 years
D	[42]	19, M	-	Japan	-	Midline	Lateral Ventricle	No reoccurrence at 5 years
D	[49]	33, M	African American	United States	Enhancing	Midline	Left Anterior Horn of Lateral Ventricle, Floor of Fourth Ventricle	No reoccurrence at 1 year
D	[47]	17, F	-	United States	Hypointense on T2, Enhancing	Midline	Right Frontal Horn of Lateral Ventricle	No reoccurrence at 1 year
E	[29]	18, M	-	United States	Well-delineated, Hyperdense, Enhancing	Midline	Diffuse. Lateral and Third Ventricles	No reoccurrence at 4 years
F	[33]	24, M	-	United States	Enhancing	-	Meninges, Diffuse Lateral, and Third Ventricles	Death
F	[34]	23, F	-	India	-	Midline	Diffuse Lateral Ventricle and Third Ventricle; Caudate, Fornix, Optic Chiasm, Optic Nerve	Death
G	Present Case	19, M	Caucasian	United States	Enhancing	Midline	Lateral and Third Ventricles	No reoccurrence at 12 months

## Conclusions

In conclusion, intracranial germinomas are uncommon tumors that primarily affect adolescent males in Asian countries. We present a rare case in a Western country of a pure germinoma in the pineal and suprasellar regions with the atypical radiographic appearance of diffuse spread to the lateral and third ventricles. Unlike other malignant germ cell tumors, germinomas have marker-negative CSF samples that are important in the normal diagnostic workup of diffuse subependymal lesions. Histopathologic correlation is required for definitive diagnosis in the United States and can be achieved with endoscopic tissue sampling. Germinomas are highly radio- and chemotherapy-sensitive and have a fair prognosis with modern therapeutic techniques. A germinoma should be considered with simultaneous midline and diffuse ventricular lesions.
